# Overexpression of SIRT1 promotes metastasis through epithelial-mesenchymal transition in hepatocellular carcinoma

**DOI:** 10.1186/1471-2407-14-978

**Published:** 2014-12-18

**Authors:** Chong Hao, Peng-Xi Zhu, Xue Yang, Zhi-Peng Han, Jing-Hua Jiang, Chen Zong, Xu-Guang Zhang, Wen-Ting Liu, Qiu-Dong Zhao, Ting-Ting Fan, Li Zhang, Li-Xin Wei

**Affiliations:** Tumor Immunology and Gene Therapy Center, Eastern Hepatobiliary Surgery Hospital, Second Military Medical University, 225 Changhai Road, Shanghai, 200438 China; Department of Pharmacy, Chang Hai Hospital, the Second Military Medical University, 168 Changhai Road, Shanghai, 200433 China

**Keywords:** SIRT1, Human hepatocellular carcinoma, Metastasis, Epithelial-mesenchymal transition

## Abstract

**Background:**

SIRT1 is a member of the mammalian sirtuin family with the ability to deacetylate histone and nonhistone proteins. The correlation between SIRT1 expression and tumor metastasis in several types of cancer has aroused widespread concern. This study investigated SIRT1 expression and its prognostic value in hepatocellular carcinoma (HCC). The function of SIRT1 in hepatocarcinogenesis was further investigated in cell culture and mouse models.

**Methods:**

Western blotting and immunohistochemistry were used to explore SIRT1 expression in HCC cell lines and primary HCC clinical specimens. The functions of SIRT1 in the migration and invasion in the HCC cell line were analyzed by infecting cells with adenovirus containing full-length SIRT1 or sh-RNA. The effect of SIRT1 on tumorigenicity in nude mice was also investigated.

**Results:**

SIRT1 expression was significantly overexpressed in the tumor tissues and HCC cell lines. SIRT1 significantly promoted the ability of migration and invasion in HCC cells. In addition, experiments with a mouse model revealed that SIRT1 overexpression enhanced HCC tumor metastasis *in vivo*. Furthermore, we demonstrated that SIRT1 significantly enhanced the invasive and metastatic potential by inducing epithelial-mesenchymal transition in HCC cells. A clinicopathological analysis showed that SIRT1 expression was significantly correlated with tumor size, tumor number, and TNM staging. Kaplan–Meier survival curves revealed that positive SIRT1 expression was associated with poor prognosis in patients with HCC.

**Conclusions:**

Our data suggest that SIRT1 may play an important role in HCC progression and could be a potential molecular therapy target for HCC.

**Electronic supplementary material:**

The online version of this article (doi:10.1186/1471-2407-14-978) contains supplementary material, which is available to authorized users.

## Background

Liver cancer is the fifth most frequently diagnosed cancer and the second leading cause of cancer deaths worldwide. In general, the highest incidence rates are estimated to occur in Asia and Africa. Of all liver cancers, hepatocellular carcinoma (HCC) is the most common primary liver malignancy in adults, accounting for 70–85% of cases [1]. Modifiable risk factors, including chronic hepatitis B virus or hepatitis C virus infection, aflatoxin B1, and alcohol are thought to be the main causes of HCC [1]. HCC is an insidious disease without pain, the diagnosis of which is usually made at a late stage; thus, excluding curative treatments and leaving patients with few therapeutic options. The high mortality from HCC is mainly attributed to the invasion pattern and intrahepatic and/or extrahepatic metastases, but the exact mechanism remains unclear. Therefore, a thorough understanding of the underlying mechanisms of tumor metastasis is vital for effective prevention and therapeutics in the field of liver cancer research.

Sirtuins in mammals share extensive homologies with the *Sirt2* gene in yeast and comprise a small family with seven members, respectively named SIRT1–SIRT7, which play a critical role in the regulation of critical biological processes such as metabolism, aging, oncogenesis, and cancer progression [2, 3]. Notably, SIRT1 is the most well-characterized member of the sirtuin family and plays a key role in both cell death and survival with other p53 family members, FOXO transcription factors, and the nuclear factor-κB family [4]. Moreover, whether SIRT1 acts as a tumor promoter or tumor suppressor remains controversial. It has been reported that SIRT1 is upregulated in a spectrum of cancers, including lymphomas, leukemia and soft-tissue sarcomas, prostate cancer, lung cancer, and colon carcinoma via one or more of these targets [5–9]. However, a significant decrease in SIRT1 expression is observed in breast cancer 1-associated breast cancer than in normal controls [10]. A high level of SIRT1 expression was detected in 40 paired HCC tissues, compared with normal tissue, suggesting that SIRT1 may play a role in telomeric maintenance and genomic stability [11]. The role of SIRT1 in HCC is of particular interest for developing SIRT1 as a promising therapeutic target.

In this study, we examined SIRT1 expression in HCC cell lines and human HCC tissue samples. The correlations among SIRT1 expression, clinicopathological variables, and survival time of patients with HCC were evaluated, and the role of SIRT1 in HCC prognosis was assessed. We uncovered a key role for SIRT1 as a tumor promoter that enhances invasive and metastatic potential in HCC using HCC cell models. Our findings provide a rationale for clinically exploring the use of sirtuin inhibitors in HCC therapy.

## Methods

### Cell culture

The human hepatocellular carcinoma cell lines HepG2, Huh7, Hep3B, and SMMC-7721 were obtained from the Cell Bank of Type Culture Collection of Chinese Academy of Sciences, Shanghai Institute of Cell Biology, Chinese Academy of Sciences. HepG2, Huh7, Hep3B, and SMMC-7721 cells were cultured in Dulbecco’s modified Eagle’s medium (high glucose) (Gibco, Grand Island, NY, USA) containing 10% fetal bovine serum (FBS) and 100 U/ml of penicillin/streptomycin (Gibco). All cells were incubated in a humidified incubator at 37°C with 5% CO_2_ and 95% air.

### RNA extraction and real-time quantitative PCR

Total RNA extraction, complementary DNA (cDNA) synthesis, and qPCR were performed as described previously [12]. The primer sequences used in the qPCR are shown in Table 1.

**Table 1:** **Sequences of RT-PCR oligonucleotide primers**

	Sequence (5’ → 3’)	
E-cadherin	F	TGAAGGTGACAGAGCCTCTGGA
	R	TGGGTGAATTCGGGCTTGTT
Vimentin	F	TGGCCGACGCCATCAACACC
	R	CACCTCGACGCGGGCTTTGT
Twist	F	GCCAGGTACATCGACTTCCTCT
	R	TCCATCCTCCAGACCGAGAAGG
SIRT1	F	TGCTGGCCTAATAGAGTGGCA
	R	CTCAGCGCCATGGAAAATGT
Snail	F	CCTCCCTGTCAGATGAGGAC
	R	CCAGGCTGAGGTATTCCTTG
GAPDH	F	TGCCAAATATGATGACATCAAGAA
	R	GGAGTGGGTGTCGCTGTTG

### Protein extraction and Western blotting analysis

Total soluble protein extractions from cultivated cells and Western blot analyses were performed as described previously [12]. Antibodies used for Western blotting were specific for SIRT1.

### Immunohistochemistry

After fixing the tissues in formalin and embedding them in paraffin, 4-μm sections of the 99 HCCs from both tumor and nontumor tissues were deparaffinized in xylene, rehydrated in an alcohol series, and washed in distilled water. After treatment by microwave antigen retrieval, the sections were incubated with Block serum-Free (Dako, Carpentaria, CA, USA) for 10 min at room temperature to inhibit non-specific staining. Then, the slides were incubated with anti-SIRT1 (Abcam, Cambridge, MA, USA; ab32441) antibody in a moist chamber overnight at 4°C. Peroxidase activity was detected using the enzyme substrate 3,3 N-diaminobenzidine tertrahydrochloride (DAPI). The SIRT1 expression score was based on staining intensity and the area of positive cells (0 = 0–9% of cells stained positive; 1 = 10–29% of cells stained positive; 2 = 30–69% of cells stained positive; 3 = 70–100% of cells stained positive).

### Immunofluorescence

Cells seeded on glass coverslips were fixed in 4% paraformaldehyde after a 48-h treatment and with 0.1% Triton X-100 in PBS for 15 min. The coverslips were then washed and blocked with 1% bovine serum albumin in PBS for 60 min. The slides were incubated overnight with primary antibodies, washed with PBS, and treated with secondary antibodies at 37°C for 60 min. After further washing, the slides were stained with DAPI, and images were taken using an immunofluorescence microscope with identical exposure times.

### Recombinant adenovirus construction and tumor cell infection

The SIRT1-recombined adenoviral expression vector and the control vector were constructed by the rapid BP/LR reaction in the Gateway cloning system (Invitrogen, Carlsbad, CA, USA) according to the manufacturer’s instructions. Two SIRT1 siRNA sequences were designed using the Oligoengine software and verified by nucleotide BLAST searches.

Cells (1–3 × 10^6^) grown to 50–60% confluence in 10-cm Petri dishes were transfected with adenoviral vectors, siRNA sequences, or their corresponding mock sequences using a Lipofectamine 2000 kit (Invitrogen, cat. 11668–019) with the procedure provided by the manufacturer. The cells were observed under a fluorescence microscope and harvested 48 h after transfection.

### Wound healing and transwell assays

Methods for the wound healing and Transwell assays have been described previously [13–15]. Cells (5 × 10^4^) were seeded on six-well plates and grown to confluency. Then, the cells were gently scratched with a cell scraper (1.2-mm width) to create a mechanical wound. Images were taken at 0, 48 and 72 h using a phase-contrast microscope. Experiments were carried out in triplicate wells for three independent experiments. For the Transwell assay, Boyden chambers (8-μm pore size) were coated with 200-μl Matrigel at 200 μg/ml and incubated overnight. Cells (5 × 10^4^) that were seeded in medium without serum were plated in the upper chamber, and medium containing 10% FBS was added to the lower chamber as a chemoattractant. After a 48 h incubation at 37°C, the cells that migrated to the underside of the membrane were fixed in 4% formaldehyde and stained with crystal violet dye, and enumerated under a microscope. Three invasion chambers were used per condition. The values obtained were calculated by averaging the total number of cells from three filters.

### Nude mouse splenic vein metastasis assay

All experimental procedures involving animals were performed in accordance with the institutional ethical guidelines from the Animal Ethics Committee of the Second Military Medical University. Cells were injected into the splenic vein of 6-week-old nude mice (BALB/c strain) at 5 × 10^5^ cells/injection site. The mice were sacrificed after 7 weeks, and surface liver metastases were enumerated. The experimental procedures was approved by the Ethics Committee of the Second Military Medical University.

### Patients and tissue specimens

Tumors and corresponding nontumorous liver tissues along with complete clinical and pathological data were obtained from 99 patients who underwent surgical resection for HCC at the Eastern Hepatobiliary Surgery Hospital between 1997 and 2007. Patients had not received any adjuvant therapy before surgery. Informed consent was obtained from each patient in this study, which was approved by the Ethics Committee of the Second Military Medical University. The Ethics Committee of the Second Military Medical University approved our collection of liver tissues for research purposes. Patient age at initial diagnosis, sex, HBV infection, presence of cirrhosis, serum total bilirubin, serum α-fetoprotein, prothrombin time, size of the tumor, tumor number, follow-up time, and disease-free and overall survival were determined. Tumor stage was determined according to the American Joint Committee on Cancer (AJCC) tumor-node-metastasis stage (TNM). All specimens were subjected to immunohistochemical evaluation.

### Statistical analysis

SIRT1 expression in HCC and nontumoral liver tissues was compared using a paired Student’s *t*-test. The correlation between SIRT1 and individual clinicopathologic parameters was evaluated using the nonparametric chi-square test. The Kaplan–Meier method was used to estimate survival rates for SIRT1 expression. Equivalences of the survival curves were tested by log-rank statistics. All statistical analyses were carried out using SPSS ver. 20.0 (SPSS, Inc., Chicago, IL, USA). A *P*-value < 0.05 was considered to indicate significance.

## Results

### Increased SIRT1 expression promoted migration and invasion in HCC cell lines

**Figure 1:**
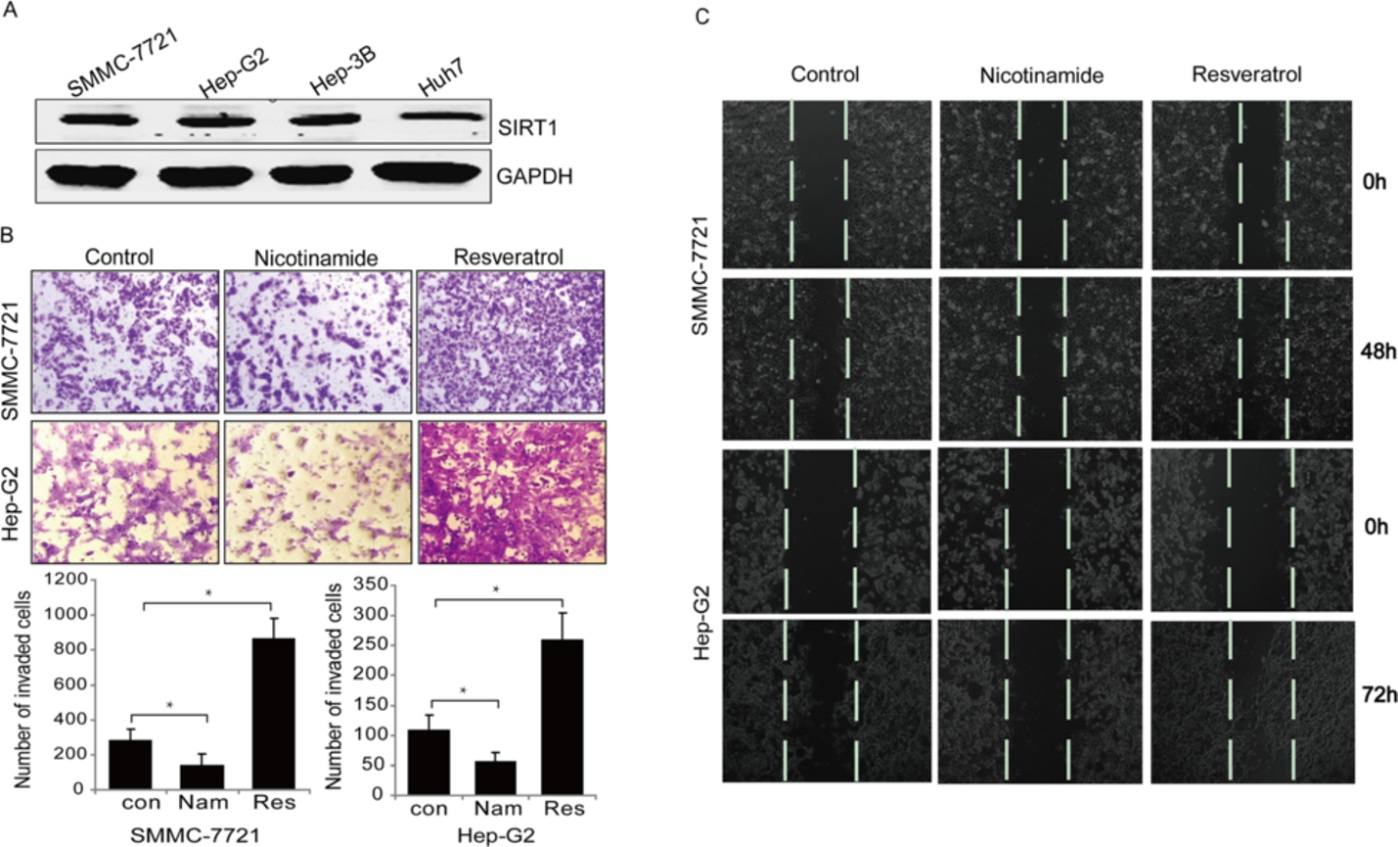
**Effects of SIRT1 activity on migration and invasion of hepatocellular carcinoma (HCC) cell lines. (A)** SIRT1 protein levels were higher in all HCC cell lines. **(B)** Invasiveness of cells was determined using the Transwell assay. SMMC-7721 and HepG2 cells were treated with nicotinamide (10 mM) or resveratrol (50 μmol/L) for 48 or 72 h. Cells were then plated in the upper chamber of the Transwell and allowed to grow for 48 h in serum-free medium, while 10% fetal bovine serum was placed in the lower chamber. The number of cells that invaded through the Matrigel was counted in 10 fields under a × 20 objective lens and is shown as the mean ± standard deviation (*P < 0.05). **(C)** A wound healing assay was employed to determine migration of HepG2 and SMMC-7721 cells in response to an SIRT1 inhibitor or activator. Cells were monitored every 24 h for 2 days to evaluate the rate of migration into the scratched area. **(B)** and **(C)** are representative of at least three independent experiments (×200).

We determined SIRT1 expression using a panel of HCC cell lines. Western blotting results showed that the SIRT1 protein was overexpressed in the HCC cell lines (SMMC-7721, HepG2, Hep3B and Huh-7; Figure 1A). Next, we evaluated whether SIRT1 plays a role in migration and invasion of HCC cells. An earlier study suggested that resveratrol, a phytoalexin found in grapes, mimics the effect of caloric restriction, which has been speculated to be mediated through activation of SIRT1 [16]. Nicotinamide, a SIRT1 inhibitor, inhibits SIRT1 deacetylase activity without affecting SIRT1 protein levels [17]. We carried out a scratch wound-healing and Matrigel invasion assay in SMMC-7721 and HepG2 cells. A clear effect on motility and invasion were observed in the presence of resveratrol (50 μmol/L) for 48 or 72 hr separately in both cell lines. In contrast, invasion and migration were inhibited significantly by nicotinamide (10 mM; Figure 1B, C, *P* < 0.05). Besides that, we measured the effect of resveratrol (50 μmol/L) on the cell viability and apoptosis of HCC cells. The results demonstrated that resveratrol could slightly inhibit the cell viability and enhance apoptosis (Additional file 1: Figure S1).

**Figure 2:**
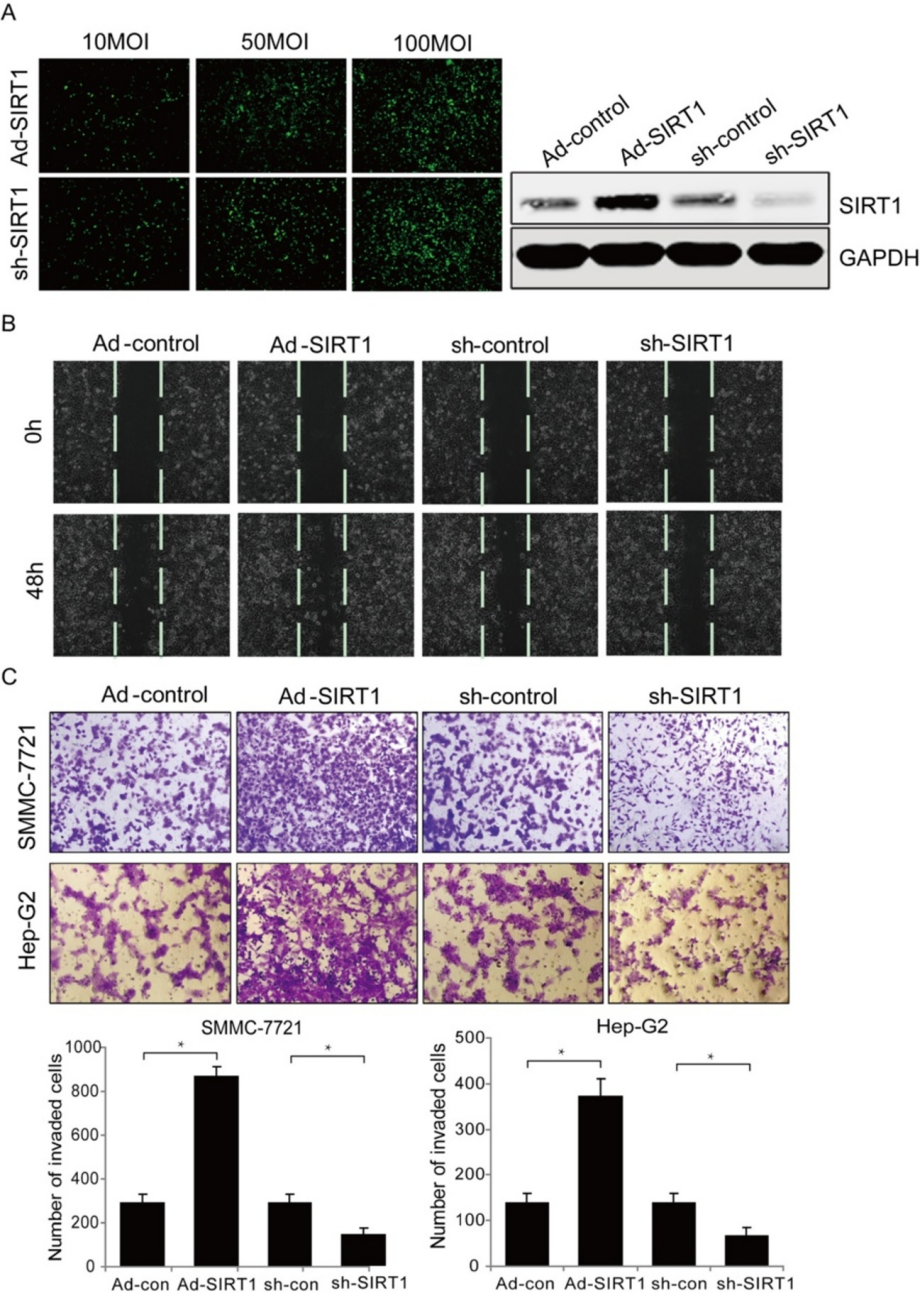
**Overexpression of SIRT1 promotes hepatocellular carcinoma (HCC) cell migration and invasion. (A)** SMMC-7721 cells were transfected with adenoviral vectors, siRNA sequences, or their corresponding mock sequences to knockdown or overexpress SIRT1 at the protein level. **(B)** and **(C)** SMMC-7721 and HepG2 cells were transduced with adeno-associated virus to express SIRT1 or siRNA to knockdown SIRT1. Migration of pretreated HCC cells was determined by wound healing assay (A, ×200) and the invasiveness of the pretreated HCC cells was determined by Transwell assay (B, ×200, *P < 0.05).

Based on the inhibitor/activator treatment, we constructed adenovirus-mediated shRNA knockdown and overexpressed SIRT1 to elucidate the cellular functions at the protein level (Figure 2A). We found that SIRT1 (shSIRT1) depletion markedly diminished wound-healing capacity and impaired cell invasion through Matrigel (Figure 2B, C, *P* < 0.05), whereas SIRT1 (Ad-SIRT1) overexpression enhanced cell migration and invasion. Taken together, these data suggest that SIRT1 promotes migration and invasion by HCC cells *in vitro*.

### Inhibiting SIRT1 decreases hepatic tumorigenesis in mice

**Figure 3:**
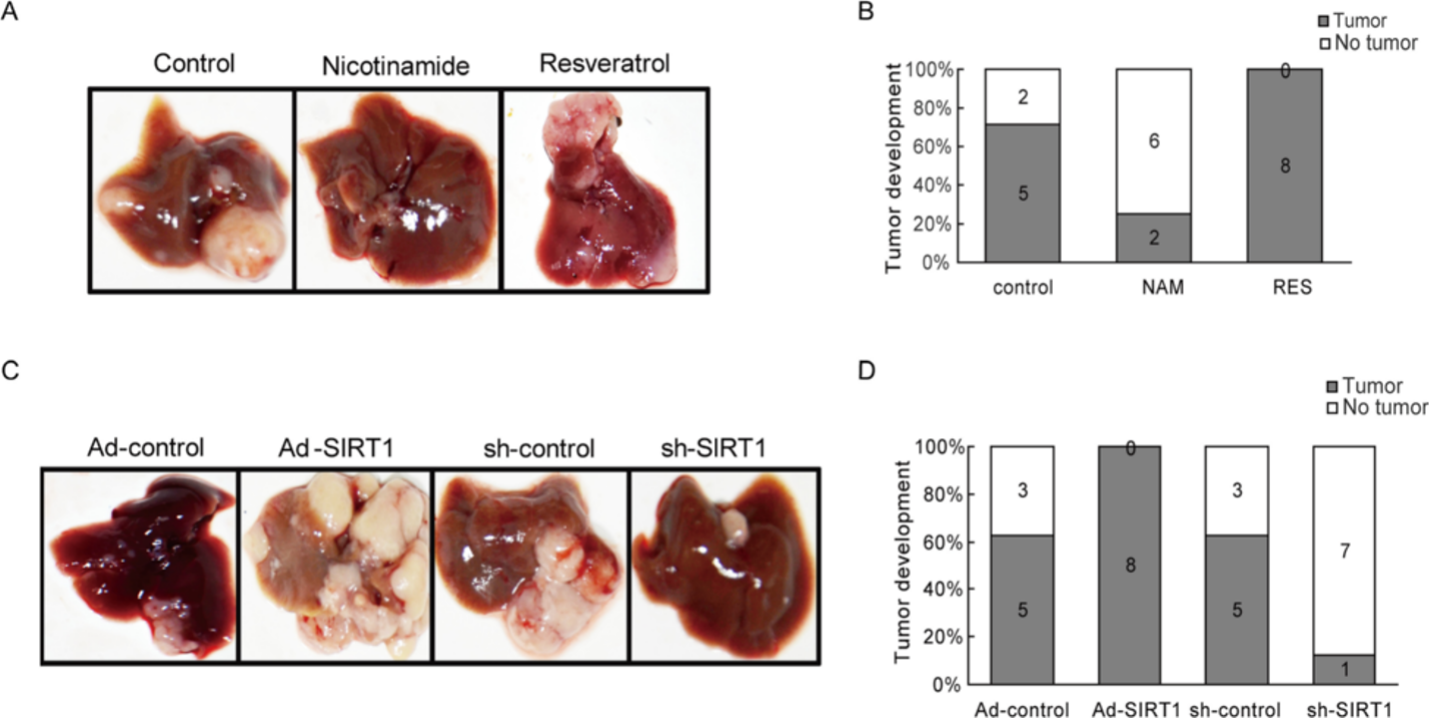
**SIRT1 promotes hepatocellular carcinoma (HCC) tumorigenicity** ***in vivo*** **. (A)** and **(C)** Photographs of metastatic liver nodules in nude mice by splenic-vein injection of SMMC-7721 cells untreated or treated with nicotinamide (50 μM), resveratrol (25 mg/mL), Ad-SIRT1, Ad-control, sh-SIRT1, or sh-control. **(B)** and **(D)** The rate of occurrence of metastatic nodules was quantified in nude mouse livers (n = 8 per group). Values for individual mice are shown above the bars (*P < 0.05).

Based on our *in vitro* experiments, the effect of SIRT1 in an ectopic model of liver cancer metastasis was subsequently tested. We chose SMMC-7721, the most invasive HCC cell line, which has been employed in *in vivo* metastasis assays with nude mice [18]. We injected SMMC-7721 cells pretreated with nicotinamide (50 μM), resveratrol (25 mg/mL), or a vehicle control into the spleen of each mouse. Six weeks later, a necropsy was performed to determine tumor growth and metastatic pattern. Two of eight mice in the nicotinamide group developed smaller liver surface metastatic nodules compared with six of eight mice in the control group; however, mice injected with resveratrol-treated SMMC-7721 cells had almost all metastatic nodules (Figure 3A, B). The rate of occurrence of metastatic nodules in the liver decreased dramatically in the sh-SIRT1 group when compared with that in the Ad-SIRT1 group (Figure 3C, D). These results indicate that SIRT1 has effects on the metastatic potential of HCC cells *in vivo*.

### SIRT1 overexpression promotes HCC cell metastasis by regulating the epithelial-to-mesenchymal transition (EMT) in HCC

**Figure 4:**
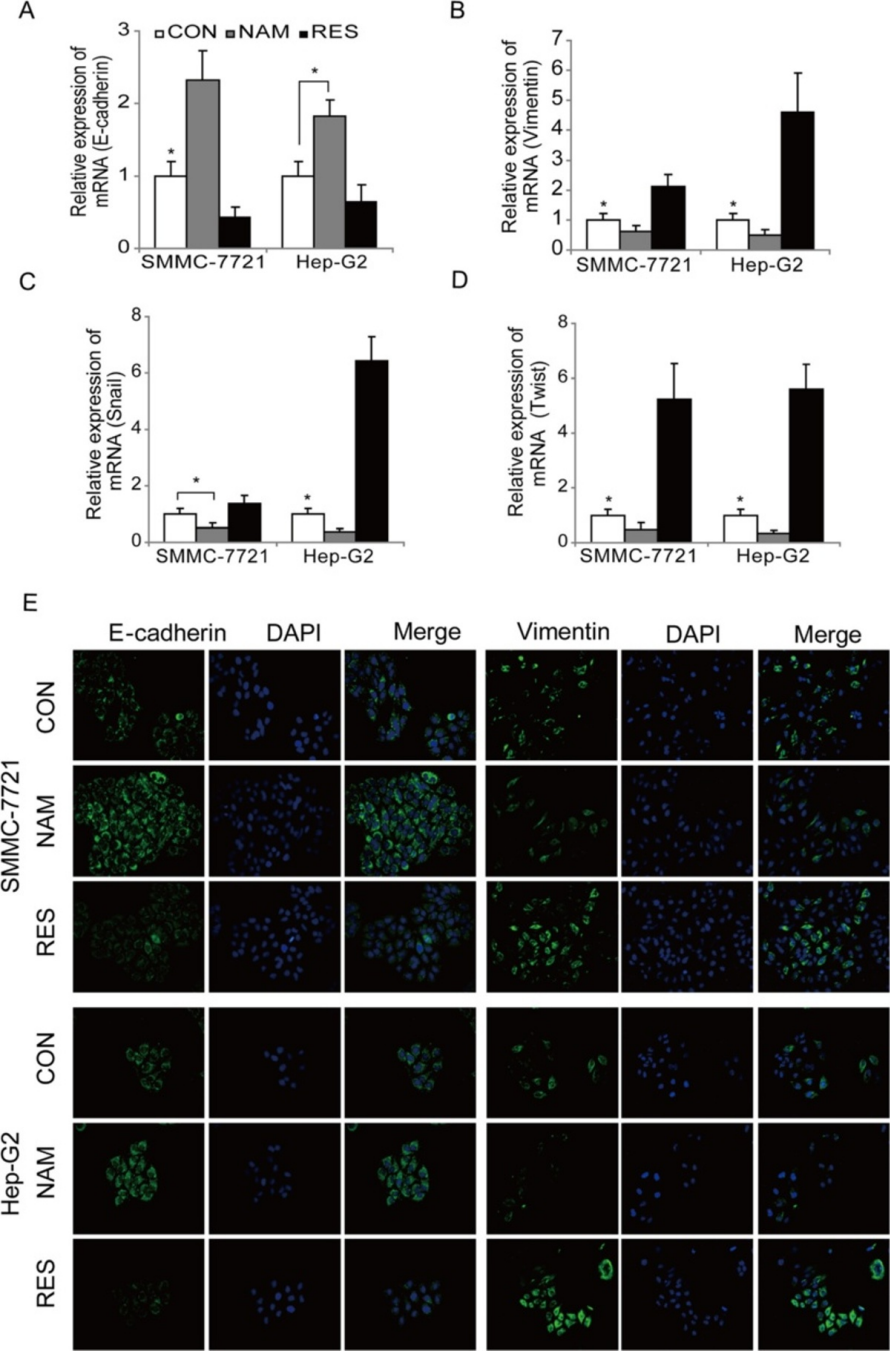
**SIRT1 induces epithelial-mesenchymal transition (EMT) in hepatocellular carcinoma (HCC) cells. (A) - (D)** RT-PCR was used to detect changes in the expression of EMT-associated genes in HCC cells treated with nicotinamide (NAM) or resveratrol (RES). Results represent means of triplicate experiments ± standard errors (*P < 0.05). **(E)** Immunofluorescence staining for E-cadherin and vimentin was performed in HepG2 and SMMC-7721 cells that were either untreated or treated with NAM or RES. Nuclei were counterstained with DAPI (×200).

Growing evidence indicates that the EMT is correlated with HCC cell invasion and metastasis [19]. The EMT is regulated by several transcription factors, such as Snail, Slug, Twist, and Zeb1, each of which binds to the E-cadherin promoter and repress transcription [19]. To further understand whether SIRT1 promotes liver cancer invasion and migration by EMT, we performed a reverse transcription-polymerase chain reaction analysis of the expression of an epithelial marker (E-cadherin), mesenchymal markers (vimentin) and EMT-associated transcriptional factors (Snail and Twist). Consistent with resveratrol promoting SMMC-7721 cell invasion and migration, E-cadherin was downregulated, whereas vimentin, Snail, and Twist were upregulated (Figure 4A-D, *P* < 0.05). In contrast, nicotinamide yielded the opposite result, suggesting the potential of reversal of the EMT. This also occurred in HepG2 cells. The E-cadherin and vimentin immunofluorescence results further supported the finding that SIRT1 promotes EMT-mediated HCC cell invasion and metastasis (Figure 4E).

### SIRT1 was detected in HCC tissues and was associated with the progression and prognosis of HCC

**Figure 5:**
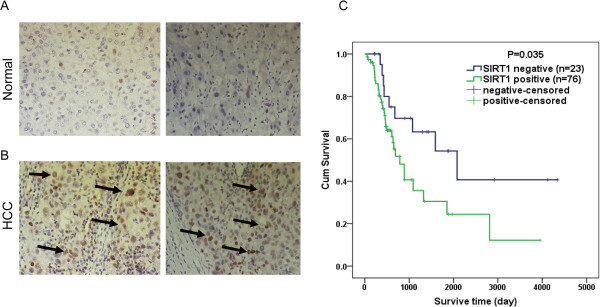
**Immunohistochemical analysis of SIRT1 expression in primary hepatocellular carcinoma (HCC) surgical specimens. (A)** and **(B)** Nuclear expression of SIRT1 was negative in 23/99 of HCC tissues **(A)**, and positive in 76/99 **(B)** (400 × magnification in A–B). **(C)** The patients with HCC were divided into positive- and negative-expression groups based on SIRT1 immunostaining scores. The survival rate of the patients in the positive-expression group was significantly lower than that of patients in the negative-expression group (*P* = 0.035 by the log-rank test).

We first examined SIRT1 expression levels in clinical HCC specimens. Immunohistochemical staining results showed that SIRT1 expression in HCC specimens was significantly upregulated compare with that in the adjacent non-tumoral liver tissue. SIRT1 overexpression was observed in 76 of 99 (76.8%) HCC specimens when compared with the non-malignant group of HCC cells (37 of 99, 37.4%). All 99 patients with HCC were divided into a positive expression group (n = 76) and a negative expression group (n = 23) (Figure 5A, B).

Significant associations were observed between SIRT1 expression and size of the tumor, tumor number, and TNM stage (*P* = 0.043, *P* = 0.031, *P* = 0.038, respectively) for the 99 patients with HCC. Relationships between SIRT1 expression and clinicopathological features of the patients with HCC are summarized in Table 2. In addition, patients with HCC in the negative SIRT1 expression group showed a favorable survival outcome (*P* = 0.035), with mean and median survival times of 80 and 69 months, respectively compared with those of the positive expression group (45 months and 26 months). The SIRT1 expression survival curves in tumors are shown in Figure 5C.

**Table 2:** **Relationship between SIRT1 expression and the clinicopathological features of 99 patients with primary hepatocellular carcinoma**

Factor	Category	Total No. of patients	SIRT1 negative (n = 23)	SIRT1 positive (n = 76)	***P***
Age (years)	>50	44	8 (34.8)	36 (47.4)	0.287
	≤50	55	15 (65.2)	40 (52.6)	
Gender	Male	89	22 (95.7)	67 (88.2)	0.296
	Female	10	1 (4.3)	9 (11.8)	
Size of tumor (cm)*	>5	44	6 (26.1)	38 (50.0)	0.043
	≤5	55	17 (73.9)	38 (50.0)	
Tumor number*	Single	68	20 (87.0)	48 (63.2)	0.031
	Multiple	31	3 (13.0)	28 (36.8)	
TBIL (μmol/L)	>20	23	4 (17.4)	19 (25.0)	0.449
	≤20	76	19 (82.6)	57 (75.0)	
HBsAg	Positive	96	22 (95.7)	74 (97.4)	0.674
	Negative	3	1 (4.3)	2 (2.6)	
AFP (ng/ml)	>400	40	7 (30.4)	33 (44.6)	0.228
	≤400	57	16 (69.6)	41 (55.4)	
Liver cirrhosis	Absent	35	9 (39.1)	26 (34.2)	0.665
	Present	64	14 (60.9)	50 (65.8)	
TNM*	I	47	17 (73.9)	30 (39.5)	0.038
	II	18	2 (8.7)	16 (21.1)	
	III	16	2 (8.7)	14 (18.4)	
	IV	18	2 (8.7)	16 (21.1)	

## Discussion

One study revealed that SIRT1 regulates critical biological processes, including metabolism, aging, DNA damage, apoptosis, and cancer progression [20]. However, it remains controversial whether SIRT1 acts as a tumor promoter or suppressor due to the temporal and special distribution of different SIRT1 upstream and downstream targets and factors in different tissue contexts [21]. SIRT1 is overexpressed in human prostate cancer, acute myeloid leukemia, and primary colon cancer [9, 22, 23]. SIRT1 expression is decreased in many other types of cancers, including human bladder, glioblastoma, and ovarian cancer [24] compared to corresponding normal tissues. Thus, it is difficult to clarify the role of SIRT1 in tumorigenesis, as this protein functions in a cell- or tissue-related fashion.

The role of SIRT1 in tumorigenesis is controversial. Our study showed that SIRT1 acted primarily as a promoter of the migration and invasion of HCC cells. We selected two HCC cell lines (SMMC-7721 and HepG2) and carefully evaluated the direct effect of SIRT1 on their invasive and metastatic potential. We utilized a SIRT1 inhibitor and sh-SIRT1 to suppress expression, which also suppressed HCC progression. Restoring SIRT1 expression significantly promoted cell invasion and migration. In parallel experiments, we found that SIRT1 overexpression promoted tumor growth in an injectable mouse model. Finally, we demonstrated that SIRT1 significantly enhanced the invasive and metastatic potential of HCC cells by inducing EMT.

EMT is a process by which the mesenchymal phenotype is acquired by epithelial cells, which is a key process in a variety of human epithelial tumors. EMT is associated with liver cancer migration and metastasis. We speculated that EMT-associated transcription factors might be target genes of SIRT1 in HCC. Our data suggested that SIRT1 play a role in EMT. This observation was supported by SIRT1 activity in HCC cells with reverse EMT features, including a change in the expression of mesenchymal and epithelial markers. Consistent with our results, a previous study showed that silencing SIRT1 in human prostate cancer cells decreases cell migration *in vitro* and metastasis *in vivo* in immunodeficient mice, which is significantly independent of the effects of SIRT1 on prostate cancer growth and survival mediated by inducing EMT [25]. Byles et al. confirmed that SIRT1 functions as a corepressor with ZEB1 specifically to suppress E-cadherin transcription and upregulate mesenchymal markers during the EMT in prostate cancer [25]. Zhang et al. showed that upregulation of miR-204 influences EMT-associated genes expression in gastric cancer cells, which are involved in post-transcriptional repression of SIRT1 [26]. These findings strengthen the hypothesis that SIRT1 acts as an HCC tumor promoter.

In this study, we found that SIRT1 expression was high in most primary HCC tumor tissues compared with that in corresponding non-tumorous liver tissues by immunohistochemical analysis. Consistent with these observations, Western blotting showed that the SIRT1 protein was overexpressed in HCC cell lines. We further used a relatively large series of clinical tissue samples to analyze the effect of SIRT1 expression level on the clinical and pathological features of primary HCC. We presume that SIRT1 protein expression in HCC was significantly associated with the pathological features of tumor size, tumor number, and TNM stage. In addition, our Kaplan–Meier survival analysis revealed that SIRT1 expression was significantly linked to a poor prognosis after surgical resection in patients with HCC (*P* = 0.035), suggesting that SIRT1 can serve as a new predictor of prognosis in patients with HCC after surgical resection. Taken together, these results indicate that SIRT1 may play a role in HCC development.

In conclusion, we found that SIRT1 expression was upregulated in the majority of HCC clinical tissue specimens and that SIRT1 expression may be correlated with an unfavorable prognosis in patients with HCC. Cell culture studies confirmed that SIRT1 overexpression promoted cell invasion and migration in HCC cell lines. The mouse model experiments revealed that SIRT1 overexpression significantly enhanced metastatic nodules. Our results indicate that SIRT1 may enhance the invasive and metastatic potential of HCC cells by inducing the EMT. These findings provide information that will facilitate development of a novel therapeutic approach against HCC metastasis.

## Conclusions

Our results indicate that SIRT1 may enhance the invasive and metastatic potential of HCC cells by inducing EMT. This provides information that will facilitate development of a novel therapeutic approach against HCC metastasis.

## Electronic supplementary material

Additional file 1: Figure S1: Sequences of RT-PCR oligonucleotide primers. (PDF 256 KB)

## Abbreviations

PBS: Phosphate-buffered saline
HCC: Hepatocellular carcinoma
EMT: Epithelial to mesenchymal transition
FBS: Fetal bovine serum
RES: Resveratrol
NAM: Nicotinamide
IHC: Immunohistochemistry.
