# Supplementary material for: Overexpression of SIRT1 promotes metastasis through epithelial-mesenchymal transition in hepatocellular carcinoma
Source: BMC Cancer. 2014 Dec 18;14:978. doi: 10.1186/1471-2407-14-978 (PMC4391082; doi:10.1186/1471-2407-14-978)
Supplement: Supplementary file 1 — Additional file 1: Figure S1: Sequences of RT-PCR oligonucleotide primers. (PDF 256 KB) [file 12885_2014_5245_MOESM1_ESM.pdf]

Additional file 1.

Sequences of RT-PCR oligonucleotide primers.

Gene		Sequence (5' → 3')
E-cadherin	F	TGAAGGTGACAGAGCCTCTGGA
	R	TGGGTGAATTCGGGCTTGTT
Vimentin	F	TGGCCGACGCCATCAACACC
	R	CACCTCGACGCGGGCTTTGT
Twist	F	GCCAGGTACATCGACTTCCTCT
	R	TCCATCCTCCAGACCGAGAAGG
SIRT1	F	TGCTGGCCTAATAGAGTGGCA
	R	CTCAGCGCCATGGAAAATGT
Snail	F	CCTCCCTGTCAGATGAGGAC
	R	CCAGGCTGAGGTATTCCTTG
GAPDH	F	TGCCAAATATGATGACATCAAGAA
	R	GGAGTGGGTGTCGCTGTTG

Supplementary figure 1.

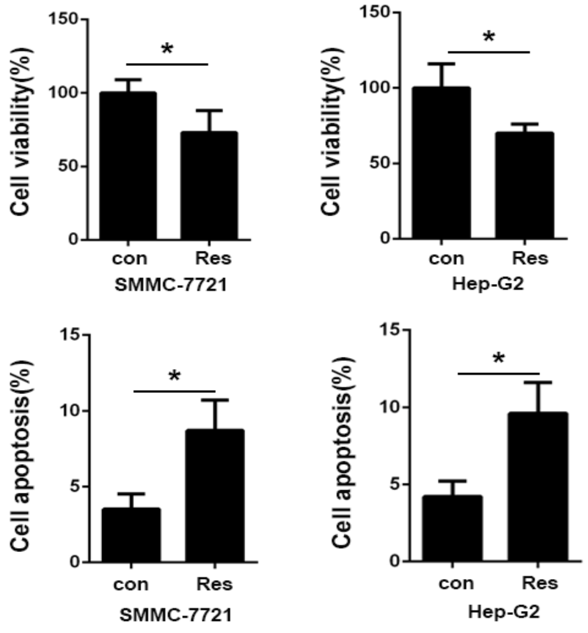

The effect of resveratrol on the cell viability and apoptosis of HCC cells were measured using cell viability assay and apoptosis assay. SMMC-7721 and HepG2 cells were treated with

resveratrol (50 μmol/L). Results represent means of triplicate experiments ± standard errors (*$P < 0.05$).
